# Assessment of s-nitrosothiol and thiol/disulfide levels in acute coronary syndrome patients

**DOI:** 10.55730/1300-0144.5529

**Published:** 2022-11-01

**Authors:** Mehmet Murat YİĞİTBAŞI, Abdullah Nabi ASLAN, Harun KUNDİ, Mücahit Furkan ERKILIÇ, Özcan EREL, Hacı Ahmet KASAPKARA

**Affiliations:** 1 Department of Cardiology, Faculty of Medicine, Ankara Yıldırım Beyazıt University, Ankara, Turkey; 2 Department of Cardiology, Cardiovascular Hospital, Ankara City Hospital, Ankara, Turkey; 3 Department of Biochemistry, General Hospital, Ankara City Hospital, Ankara, Turkey; 4 Department of Biochemistry, Faculty of Medicine, Ankara Yıldırım Beyazıt University, Ankara, Turkey

**Keywords:** Acute coronary syndrome, ST segment elevation myocardial infarction, non-ST-elevation myocardial infarction, S-nitrosothiol, thiol, disulfide

## Abstract

**Background/aim:**

The level of nitric oxide (NO) is important to protect the heart from ischemic damage in acute coronary syndrome (ACS) patients. S-nitrosothiol (SNO) is a molecule that represents the main form of NO storage in the vascular structure. In addition, dynamic thiol/disulfide homeostasis (TDH) is known to play an important role in maintaining the oxidant-antioxidant balance. In this study, our aim is to evaluate the oxidative/nitrosative stress status according to SNO level and TDH in patients with ACS.

**Materials and methods:**

The study included 124 patients who were admitted to the emergency service and 124 consecutive individuals who applied to the cardiology outpatient clinic with cardiac complaints and underwent coronary angiography (CAG). Blood was drawn from all participants included in the study to determine SNO, nitrite, total thiol, native thiol, and disulfide levels after 12 h of fasting.

**Results:**

Serum SNO levels were found to be significantly lower in ACS patients compared to the control group (0.3 ± 0.08 vs. 0.4 ± 0.10 μmol/L, successively, p < 0.001). In addition, while the total thiol, native thiol, and native thiol/total thiol levels were lower in the patient group compared to the control group, nitrite, disulfide/native thiol and disulfide/total thiol levels were higher. As a result of multivariate logistic regression analysis, it was determined that age, gender, smoking, low-density lipoprotein cholesterol, glycosylated haemoglobin, and SNO levels were independent predictors in predicting ACS patients.

**Conclusion:**

S-nitrosothiol and thiol levels were found to be significantly lower in ACS patients. In addition, SNO molecule was independently associated with the presence of ACS diagnosis.

## 1. Introduction

Acute coronary syndrome (ACS) refers to a group of clinical entities that include ST-elevation myocardial infarction (STEMI), non-ST-elevation myocardial infarction (NSTEMI), and unstable angina. In 90% of ACS cases, the main underlying cause is atherosclerosis which is associated with plaque formation consisted of fatty deposition in arterial walls.

Oxidative stress (OS) has been reported as one of the underlying mechanisms of CAD [1,2]. Thiols are important antioxidant agents in humans. Thiols, containing the sulfur analog of alcohol are found in plasma in free or oxidized form. In conditions of high OS, thiol levels drop to neutralize ROS with the sulfhydryl groups of thiols playing a critical role in ensuring this [3]. Recently, it has been reported that the thiol/disulfide ratio is a new marker of OS and may vary in ACS [4,5].

Nitric oxide (NO) is a product of endothelium and plays an important role in regulating the vascular tone of blood vessels, especially in coronary circulation. Decreased NO activity is one of the most important signs of ED [6]. The activity of NO as a cell signaling molecule via cyclic guanosine monophosphate (cGMP) is well established. Recent studies suggest that NO may modify proteins posttranslationally, via a nitrosative pathway known as S-nitrosylation [7]. S-nitrosothiols are simple organic thioesters of nitrites or functional groups containing a nitroso group covalently bonded to the sulfur atom of a thiol. NO lifetime is <2 msin blood [8] and <2 s in tissues [9]. This short lifetime is believed to be a limitation for the biological activity of NO. However, when NO complexes with thiol groups form SNO, this formation prolongs NO’s life by protecting NO from being inactivated by metalloproteins and free radicals.

In this study, we aimed to evaluate the oxidative and nitrosative stress status in ACS patients with SNO level and TDH.

## 2. Materials and methods

### 2.1. Patients and study design

This study with a cross-sectional design was carried out between September 2021 and December 2021. A total of 248 patients, 124 patients aged 18–80 years, who applied to the adult emergency department with chest pain and were diagnosed with ACS as a result of the examinations, and 124 patients between the ages of 18–80, who were similar to the patient group in terms of demographic characteristics, were included in the study.

Patients with known heart failure (HF), cardiogenic shock, sudden cardiac arrest, who underwent cardiopulmonary resuscitation before coronary angiography, who needed intravenous infusion of furosemide and received inotropic agent (dopamine, dobutamine, noradrenaline) infusion were excluded from the study.

ACS was diagnosed according to the 2017 Acute Myocardial Infarction in Patients Presenting with ST Segment Elevation European Society of Cardiology (ESC) guidelines [10] and the 2020 Acute Coronary Syndromes in Patients Presenting without Persistent ST Segment Elevation [11] ESC guidelines. Control group consisted of patients with the diagnosis of chronic coronary syndrome (CCS) who were examined at our cardiology outpatient clinic due to the cardiac complaints (such as chest pain, exertional dyspnea, palpitations, syncope) and underwent selective right and left coronary angiography (CAG) which revealed normal coronary arteries or noncritical CAD. The CCS diagnosis and decision of CAG was made according to the 2019 ESC CCSs guideline [12]. Detailed transthoracic echocardiography (TTE) was performed on all participants by an experienced cardiologist. The study protocol was approved by the research ethics committee of Ankara City Hospital and was performed according to the Declaration of Helsinki.

### 2.2. Laboratory analysis

For the fasting blood glucose (FBG), urea, creatinine, total cholesterol, low-density lipoprotein (LDL) cholesterol, high-density lipoprotein (HDL) cholesterol, triglyceride, native thiol, total thiol, disulfide, nitrite, and SNO values to be measured from the patient and control groups, 10 mL of blood was taken into a vacuum gel tube after 12 h of fasting and was left for 15 min at room temperature to coagulate. After the blood was coagulated at room temperature, it was centrifuged at 1300 g for 10 min and the serum was separated. The separated sera were transferred to Eppendorf tubes and stored at −80 °C until the study day.

S-nitrosothiol measurement was performed according to the Saville method. In this method, mercury 2 chloride (mercury II chloride/HgCl_2_) was used to liberate/separate nitrite from SNOs. Seperated nitrite was measured using the Griess reaction. Nitrite level was measured colorimetrically according to the traditional Griess reaction. The Griess method is based on the diazotization of nitrite with a primary aromatic amine (sulfanilamide) in acidic medium and the formation of a purple nitrogen product with N-(1-naphthyl) ethylenediamine hydrochloride (NED) [13]. In addition, native thiol, total thiol, disulfide, nitrite and SNO levels of all subjects in the patient and control groups were measured 12 h after CAG. Serum native and total thiol concentrations were measured by a new fully automated colorimetric method developed by Erel et al. [14]. Modified Elman reagents were used for thiol measurement. To measure the total thiol level, a reducing agent, NaHB4, was added to the serum, thereby reducing the dynamic disulfide bonds to functional thiol groups. Formaldehyde was then added and unused NaHB4 was consumed. All thiol groups were measured after reacting with DTNB (5.50-dithiobis-(2-nitrobenzoic acid). Disulfide bonds in the sample were calculated by the equation: (total thiol – native thiol)/2. Serum collected from brachial vein blood was used to measure cardiac high-sensitivity Troponin I (hs-TpI) concentration using the ADVIA Centaur TnI Ultra test (Siemens Healthieners). The laboratory reference range was <45 ng/L.

### 2.3. Echocardiographic evaluation

Transthoracic echocardiography (IE33 echocardiography system, Philips Medical Systems, Eindhoven, The Netherlands) was performed on all patients by an experienced cardiologist. Doppler echocardiography and 2-dimensional images were obtained from parasternal short and long axis, apical 4-chamber, and subcostal 4-chamber views, according to the guidelines of the American Society of Echocardiography [15]. Left ventricular systolic and diastolic functions were analyzed using standard two-dimensional (2D) echocardiography, M-mode echocardiography, pulse wave (PW) echocardiography, and tissue Doppler echocardiography. Left atrial (LA) diameter, interventricular septum (IVS) thickness, posterior wall (PoW) thickness, left ventricular end-diastolic diameter (LVEDD), and left ventricular end-systolic diameter (LVESD) were measured with 2D imaging-guided M-mode echocardiography. Left ventricular ejection fraction was calculated using the modified Simpson’s method. The diagnosis of ischemic mitral regurgitation (MR) was made in patients with ischemic heart disease with normal valve structure and classified as mild, moderate or advanced MR.

### 2.4. Coronary angiography procedure

Coronary angiography was performed radially (GE Medical Systems, Innova IGS 620, France) using the classical Judkins technique by experienced operators (>75 cases per year). Coronary angiographic recordings were taken in left-to-right oblique projections with cranial caudal angulation at a film rate of 30 frames/s. Nonionic low osmolality contrast material (Biemexol-350 mg/mL) was used for the procedures. Coronary flow rates were determined using the TIMI frame count method. This method involves calculating frame counts. Contrast is acquired at 30 frames/s until it reaches the distal markers given for each coronary artery. The severity of the coronary stenosis was determined by measuring visually and also with the help of a caliper. Percent stenosis was calculated by measuring the lumen diameter at the point of greatest constriction and in the adjacent normal-appearing segment of the coronary artery. The stenosis was measured as a percentage of the occluded lumen diameter. A critical coronary artery stenosis was defined as a narrowing of 50% or more in the left main coronary artery and 70% or more of the lumen in the other main coronary arteries. Noncritical coronary artery stenosis was defined as stenosis affecting less than 50% of the diameter.

All statistical analyzes were performed using Stata (version 16.0 MP; StataCorp). The distribution of continuous variables was determined using the Kolmogorov-Smirnov test. Normally distributed continuous data were presented as mean ± standard deviation. Categorical data were defined as the number and percentage of cases. Variables with a statistically significant difference and a normal distribution were compared. Student’s t-test was used for two different groups with normal distribution. Pearson chi-square test was used for categorical variables. A univariate logistic regression model was constructed for each variable to show significant predictors of ACS patients, and then those with p < 0.10 were tested using a multivariate logistic regression model. The results of the multivariate regression analysis were presented as odds ratios (OR) of independent predictors of ACS and their 95% confidence intervals (CI). Receiver operating characteristics (ROC) curve analysis was used to demonstrate the discrimination performance of the final model. Finally, a nomogram with significant predictors was plotted as a graph. P-value <0.05 was considered significant.

## 3. Results

Of the 124 ACS patients included in the study, 73 (58.8%) were STEMI and 51 (41.2%) were NSTEMI patients. Since there was no patient with a diagnosis of unstable angina pectoris (USAP) who applied to our emergency department at the time of our study, USAP patients were not included. STEMI patients were grouped according to the type of MI; 41 (56.1%) had inferior MI, 29 (39.7%) had anterior MI, 2 (0.02%) had lateral MI and 1 (0.01%) had high lateral MI.

The baseline characteristics of all individuals, patients, and the control group participating in the study are shown in Table 1. The patient group was older (p < 0.001). The rate of smokers in the patient group was significantly higher (p < 0.001). In addition, chronic kidney failure (CKD) and HF rates) were found to be significantly higher in the patient group compared to the control group (p < 0.05). There was no significant difference between the two groups in terms of the incidence of DM, HT, HL, and COPD.

**Figure 1:**
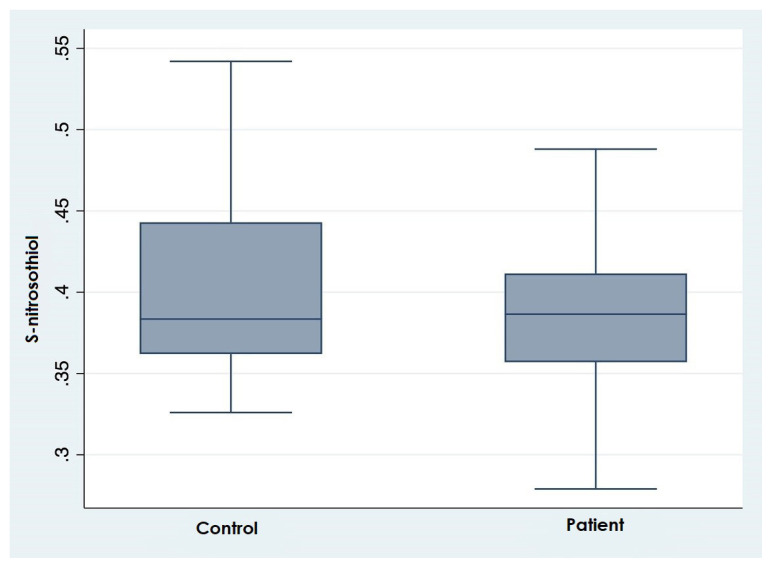
Boxplots showing S-nitrosothiol levels between patient and control groups.

The biochemical and hematological laboratory values of the entire study group, patient and control groups are presented in Table 2. Accordingly, between the two groups, no significant difference was observed between platelet count, total cholesterol levels, and hemoglobin values. However, white blood cell count, neutrophil levels, FBG, urea, creatinine, HbA1c, hs-TpI and LDL-cholesterol levels were significantly higher in the patient group than in the control group (p < 0.05). Lymphocytes, TG and HDL-cholesterol and glomerular filtration rate (GFR) were found to be lower in the patient group. When SNO and TDH parameters, which are OS biomarkers, were examined, it was observed that the SNO level was significantly lower in the patient group (Figure 1). Also, native thiol and total thiol levels and native/total thiol ratio were also found to be lower in the patient group compared to the individuals in the control group. Moreover, nitrite levels, disulfide/native thiol and disulfide/total thiol ratios were significantly higher in ACS patients compared to participants in the control group.

The transthoracic echocardiography results, CAG results, and the treatment strategies decided in patients with ACS and individuals in the control group are detailed in Table 3. Accordingly, as expected, the left ventricular ejection fraction in ACS patients was found to be significantly lower than in the control group. In parallel, LVEDD, LVESD, PoW thickness, and LA diameter were also measured larger in the patient group than in the control group. The rate of ischemic MR was also higher in patients in the ACS group than in patients in the control group as expected.

**Figure 2:**
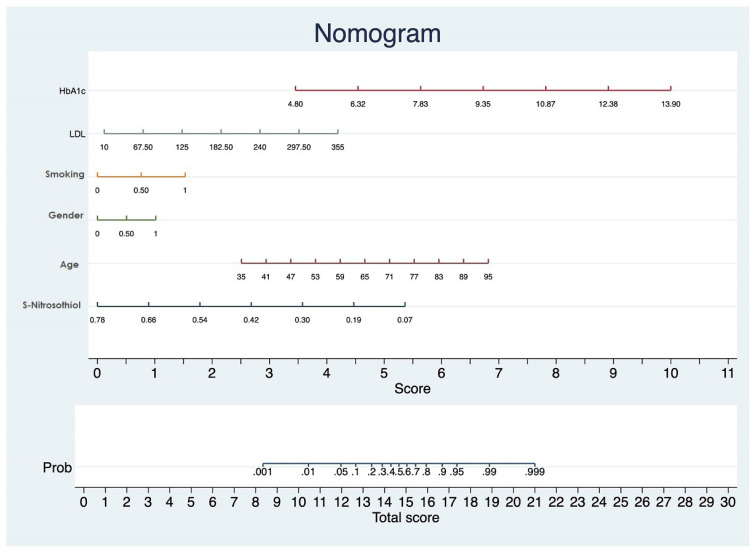
Nomogram including 6 independent variables including age, sex, smoking status, S-nitrosothiol, glycosylated haemoglobin (HbA1c and low-density lipoprotein-cholesterol (LDL) levels was created to predict the diagnosis of acute coronary syndrome (ACS).

A multivariate logistic regression model was constructed from variables including SNO, age, male sex, smoking, LDL-cholesterol, and HbA1c levels to identify independent predictors of ACS diagnosis (Table 4). In multivariate logistic regression analyzes, age, male sex, smoking, HbA1c, and LDL-cholesterol were determined as independent predictors of ACS diagnosis. Unlike previous studies, SNO was found to be an independent predictor of ACS diagnosis in this study. A nomogram was created from these 6 independent variables that could be used to predict the diagnosis of ACS (Figure 2). The importance of each variable in this nomogram is ranked according to the standard deviation across the nomogram scales. Accordingly, the value of each variable is marked according to the order in the scale and the score below is calculated. As a result, the higher the total score is calculated, the higher the probability that the person has ACS.

When the variables in the nomogram are examined one by one, it is seen that the strongest independent variables that will make the most changes in the total score are SNO and HbA1c levels. It is a well-known fact for many years that diabetes is accepted as the equivalent of CAD. Therefore, the fact that SNO level is one of the strongest independent predictors together with HbA1c is a very valuable data for our study.

**Figure 3:**
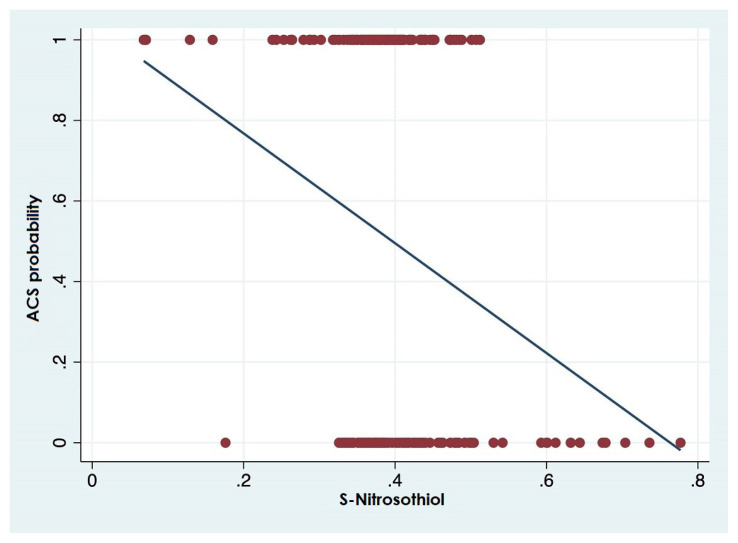
Linear diagram showing the relationship between S-nitrosothiol level and the probability of having acute coronary syndrome (ACS).

The linear diagram showing the relationship between the level of SNO and the probability of having ACS (Figure 3). The red dots in the figure represent the patient group, and it is clearly seen that the probability of having ACS decreases linearly as the SNO level increases.

**Figure 4:**
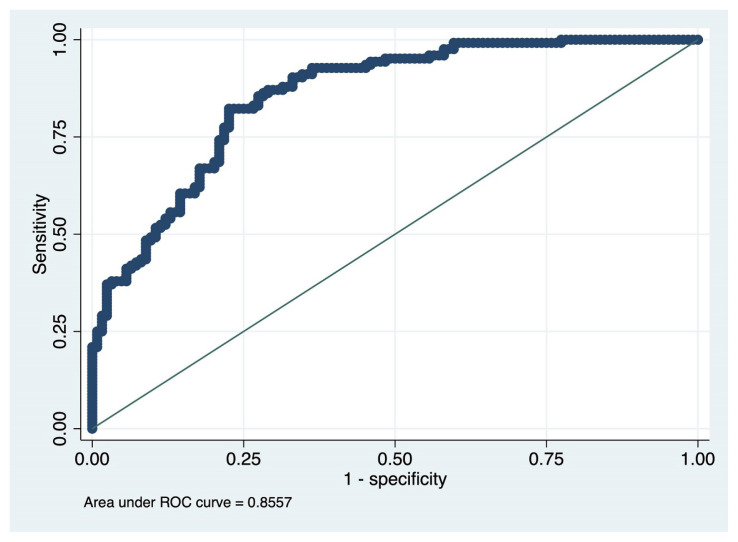
The ROC curve showing the performance of the final model in predicting acute coronary syndrome (ACS).

The ROC curve showing the performance of the final model in predicting ACS is shown in Figure 4. Accordingly, the accuracy of all these variables in predicting ACS was determined as 85% (area under the curve 0.855; 95% CI 0.839–0.953; p < 0.001). According to this model, when the probability of having ACS is evaluated according to the score of each variable in the nomogram, the percentage of the margin of error that will occur can also be revealed. For example, if we assume that the rate of SNO in predicting ACS alone is 65% relative to the nomogram, this indicates a 20% margin of error compared to 85% accuracy in the final model.

## 4. Discussion

Our study suggested that SNO levels were significantly reduced in both STEMI and NSTEMI patients and more importantly probability of having ACS increased linearly as the SNO levels decreased. In addition, it was determined that thiol levels were significantly lower and disulfide/thiol ratio was higher in ACS patients compared to individuals with noncritical coronary stenosis.

Oxidative stress is an imbalance in favor of increased reactive oxygen species (ROS) production and/or a decrease in body’s innate antioxidant defense systems [16]. OS participates in the pathogenesis of atherosclerosis and risk factors and increases free radical production in the arterial wall [17]. The major antioxidant systems in the vascular wall are superoxide dismutases, glutathione peroxidases, catalases, paraoxonases, thioredoxins, and NOs [18]. Nitric oxide is a gaseous signaling molecule produced by nitric oxide synthase in the vascular endothelium as well as in red blood cells [19]. Due to its role in inhibition of platelet aggregation and neutrophil adhesion, appropriate NO levels are important in protecting the heart from ischemic damage [20]. Also, NO is considered a potent vasodilator and can improve blood flow during reperfusion. However, NO is deactivated within seconds of its production. Its biological half-life can vary between about 6–50 s [21]. This time is estimated to be about 3–6 s in tissue and 1–2 s in blood [22]. NO is converted to nitrite and nitrate in the blood. Most of the nitrate produced is excreted by the kidneys, but a smaller part of the blood is converted to nitrite by bacteria in the oral cavity. In this way, some of the nitrite coming into the stomach turns into nitrogen gas and disappears; nitrate reaching the intestine from the blood and stomach is partially reduced to ammonia via nitrite and reabsorbed and converted to urea and excreted from the body. In this way, it is excreted from the body [23]. In this study, the reason why the nitrite level was found to be significantly higher in the patient group may be due to the catabolism of excessively increased NO. NO creates its effects through three different chemical reactions: These effects are; nitrosylation, nitrosation, and nitration. Both endogenous and exogenous NO react with thiols in proteins such as albumin to form SNOs with a longer lifetime than NO with vasodilatory activity [24]. Although the cardioprotective effects of NO via the dependent cGMP pathway are known, recent studies suggest an association between increased SNO formation and cardioprotection [10]. A study by Lima et al. [25] in mice showed that endogenous SNOs offer striking protection against myocardial ischemic injury, and this effect may be due to S nitrosylation of the transcription factor hypoxia-inducible factor-1, which is found in the hearts of mice under normoxic conditions. In cardiomyocytes, S-nitrosylation occurs in multiple proteins [26]. When nitrosothiols such as S-nitrosocysteine, S-nitrosoglutathione or S-nitrozoalbumin come into contact with cells, they release their NOs. The significantly lower SNO level in ACS patients in this study may be due to excessively secreted NOs.

Thiols are a class of endogenous molecules that are protective against damage caused by free radicals. Under OS, thiols can form reversible disulfide (RSRR) bonds. As a result of this dynamic TDH; antioxidant protection, detoxification, signal transduction, apoptosis, regulation of enzymatic activity, regulation of transcription factors, and regulation of cellular signaling mechanisms can be maintained. In recent years, the role of dynamic TDH in the pathogenesis of cardiovascular diseases has been emphasized [27]. In this study, we found that native and total thiol levels were significantly reduced in patients with ACS and thus dynamic TDH shifted to the oxidized side.

The relationship between CAD and inflammation and OS is well-known [2]. In a previous study, it was revealed that paraoxonase and arylesterase activities, both of which are antioxidant molecules, are decreased in severe CAD and this decrease predicted the severity of CAD [28]. Some other studies have also shown that increased OS or decreased antioxidant status is associated with atherosclerotic coronary artery disorders [29,30]. The outcome of our study was similar to these studies, as expected. We showed that the thiol levels were significantly increased in the ACS group with critical coronary artery stenosis. Also, ACS patients had statistically lower thiol levels compared to individuals in the control group with noncritical coronary stenosis. These results reveal a negative relationship between thiol levels and the severity of coronary artery stenosis. Kundi et al. [4] reported an increased disulfide/thiol ratio in AMI and suggested that this ratio may be an indicator for detecting acute myocardial injury. Similarly, in our study, it was shown that the disulfide/thiol ratio increased in proportion to the severity of CAD. Sivri et al. [31] investigated TDH in NSTEMI ACS patients. Similar to the results of our study, native thiol, total thiol, and native thiol/total thiol values were lower in the patient group compared to the control group and disulfide/native thiol and disulfide/total thiol values were shown to be higher in the patient group.

There are some limitations of this study. Firstly, the sample size of our study is relatively small. Secondly, since USAP patients were not included in the study, the results cannot be generalized to all ACS patients. Thirdly, the results may not cover the whole population, as the study has a single center and nonrandomized design. Last, it would be better if we could take blood samples from patients at admission. In order to evaluate SNO level and TDH in ACS patients and to generalize the results to the whole population, larger randomized studies including USAP patients are needed.

In conclusion, this study showed that decreased SNO and thiol levels may be a pathophysiological cause that may play a role in the development of ACS. Therefore, our study may inspire future studies on the development of new strategies in the diagnosis and treatment of ACS.

## Figures and Tables

**Table 1:** Baseline characteristic features of the study population.

Variables	All (n = 248)	Control (n = 124)	Patients (n = 124)	p
Age, years	60.2 ± 11.8	57.6 ± 8.7	62.9 ± 13.8	<0.001
Sex, male, n (%)	85 (34.2 %)	52 (41.9 %)	33 (26.6 %)	0.011
Smoking, n (%)	113 (45.6 %)	38 (30.6 %)	75 (60.5 %)	<0.001
HL, n (%)	142 (57.3 %)	60 (48.4 %)	82 (66.1 %)	0.005
HT, n (%)	158 (63.7 %)	74 (59.7 %)	84 (67.7 %)	0.190
DM, n (%)	88 (35.5 %)	40 (32.3 %)	48 (38.7 %)	0.290
CAD, n (%)	47 (19.0 %)	0 (0 %)	47 (37.9 %)	<0.001
COPD, n (%)	14 (5.6 %)	5 (4.0 %)	9 (7.3 %)	0.270
CRF, n (%)	19 (7.7 %)	1 (0.8 %)	18 (14.5 %)	<0.001
HF, n (%)	65 (26.2 %)	10 (8.1 %)	55 (44.4 %)	<0.001

CAD: coronary artery disease; COPD: chronic obstructive pulmonary disease; CRF: chronic renal failure; DM: diabetes mellitus; HF: heart failure; HL: hyperlipidemia; HT: hypertension

**Table 2:** Comparison of biochemical and hematological parameters of the study population, patient, and control groups.

Variables	All (n = 248)	Control (n = 124)	Patient (n = 124)	p
WBC, 10^3^/μL	9.60 ± 4.04	7.18 ± 2.01	12.01 ± 4.12	<0.001
Neutrophil, 10^3^/μL	7.69 ± 13.24	4.27 ± 1.47	11.10 ± 18.07	<0.001
Lymphocyte, 10^3^/μL	1.91 ± 1.09	2.18 ± 0.79	1.64 ± 1.27	<0.001
Platelet, 10^3^/μL	261.1 ± 76.8	254.7 ± 61.1	267.4 ± 89.6	0.190
Hemoglobin, g/L	14.9 ± 11.4	15.1 ± 9.1	14.7 ± 13.4	0.800
HbA1c, %	6.5 ± 1.6	6.0 ± 0.9	7.0 ± 2.0	<0.001
Total C, mg/dL	191.8 ± 46.2	193.3 ± 42.3	190.3 ± 50.0	0.610
LDL-C, mg/dL	123.8 ± 40.5	116.8 ± 34.6	130.8 ± 44.7	0.006
HDL-C, mg/dL	38.6 ± 11.7	42.9 ± 12.0	34.3 ± 9.8	<0.001
Triglyceride, mg/dL	156.7 ± 86.3	177.8 ± 90.7	135.6 ± 76.5	<0.001
FBG, mg/dL	129.7 ± 66.6	104.6 ± 40.9	154.9 ± 77.2	<0.001
Urea, mg/dL	38.0 ± 18.3	32.6 ± 9.2	43.5 ± 22.9	<0.001
Creatinine, mg/dL	0.92 ± 0.52	0.83 ± 0.19	1.01 ± 0.71	0.006
eGFR, ml/dk/1.73 m^2^	85.2 ± 21.7	88.0 ± 19.1	82.3 ± 23.8	0.041
Hs-Troponin I, ng/L	6250 ± 9571	6.23 ± 2.26	12,494 ± 10,265	<0.001
Native thiol, μmol/L	275.6 ± 100.1	349.2 ± 76.9	202.0 ± 57.4	<0.001
Total thiol, μmol/L	316.8 ± 103.6	391.7 ± 78.2	241.8 ± 64.1	<0.001
Disulphide, μmol/L	20.5± 5.97	21.2 ± 7.27	19.9 ± 4.21	0.078
Disulphide/Native thiol	8.3 ± 3.17	6.4 ± 3.10	10.2 ± 1.79	<0.001
Disulphide/Total thiol	7.0 ± 2.29	5.5 ± 2.18	8.4 ± 1.25	<0.001
Native/Total thiol	85.9 ± 4.57	88.8 ± 4.35	83.0 ± 2.49	<0.001
Nitrite, mg/L	3.9 ± 2.75	3.1 ± 1.89	4.7 ± 3.22	<0.001
S-Nitrosothiole, μmol/L	0.4 ± 0.09	0.4 ± 0.10	0.3 ± 0.08	<0.001

FBG: fasting blood glucose; eGFR: estimated glomerular filtration rate; HB: haemoglobin; HbA1c: glycosylated haemoglobin; HDL-C: high density lipoprotein cholesterol; Hs: high sensitivity; LDL-C: low density lipoprotein cholesterol; Total-C: total cholesterol; WBC: white blood cell

**Table 3:** Comparison of the echocardiography and treatment results of the study population.

Variables	All (n = 248)	Control (n = 124)	Patient (n = 124)	p
LVEDD, mm	45.9 ± 8.0	44.1 ± 9.3	47.7 ± 6.0	<0.001
LVESD, mm	30.4 ± 7.0	27.6 ± 6.1	33.2 ± 6.7	<0.001
IVS, mm	1.1 ± 0.7	1.0 ± 0.1	1.2 ± 0.9	0.110
PoW, mm	1.04 ± 0.12	1.03 ± 0.11	1.06 ± 0.13	0.029
LA, mm	37.5 ± 6.6	36.0 ± 5.5	39.0 ± 7.2	<0.001
LVEF, %	52.2 ± 10.5	58.5 ± 5.4	45.8 ± 10.6	<0.001
Ischemic MR, n (%)	25 (10.1%)	0 (0.0%)	25 (20.2%)	<0.001
Treatment strategy				
Medical therapy	128 (51.6%)	124 (100%)	4 (3.2%)	<0.001
PCI	113 (45.6%)	0 (0)	113 (91.2%)	<0.001
CABG	7 (2.8%)	0 (0)	7 (5.6%)	<0.001

CABG: coronary artery bypass grefting; IVS: interventricular septum; LA: left atrium; LVEDD: left ventricular end diastolic diameter; LVEF: left ventricular ejection fraction; LVESD: left ventricular end systolic diameter; MR: mitral regurgitation; PCI: percutaneous corponary intervention

**Table 4:** Assessment of independent predictors for the diagnosis of acute coronary syndromes with multivariable logistic regresssion analysis.

Variables	OR	95% CI	p
S-Nitrosothiol, μmol/L	0.001	(0.001–0.046)	0.002
Age, years	1.081	(1.045–1.119)	<0.001
Male sex	3.037	(1.335–6.909)	0.008
Smoking	5.310	(2.432–11.596)	<0.001
LDL-Cholesterol, mg/dL	1.012	(1.004–1.021)	<0.001
HbA1c, %	2.192	(1.551–3.097)	<0.001

HbA1c: glycosylated haemoglobin; LDL: low-density lipoprotein; CI: confidence interval; OR: odds ratio
